# Case-based discussion: an unusual manifestation of diaphragmatic hernia mimicking pneumothorax in an adult male

**DOI:** 10.1186/s12245-016-0108-5

**Published:** 2016-02-29

**Authors:** Pradeep Kumar Vyas, Chintamani Godbole, Susheel Kumar Bindroo, Rajiv S. Mathur, Bharathi Akula, Nilesh Doctor

**Affiliations:** Department of Emergency and Respiratory Medicine, Jaslok Hospital and Research Centre, 15 Dr. G. Deshmukh Marg, Mumbai, India Pin-Code 91-400026; Department of Gastrointestinal-Surgery, Jaslok Hospital and Research Centre, Mumbai, India 400 026

**Keywords:** Pleural diseases, Respiratory muscles, Rare lung diseases, Lung trauma, Hernia diaphragmatic, Pneumothorax

## Abstract

Diaphragmatic hernia is an important cause of emergency hospital admission associated with significant morbidity. It usually results from congenital defect or rupture in the diaphragm due to trauma. Prompt and appropriate diagnosis is necessary in patients with this condition, as surgical intervention by either abdominal or thoracic approach may be necessary. Here, we report a case of left-sided diaphragmatic hernia presenting with sudden onset of breathlessness, respiratory distress and left-sided chest pain radiating to the abdomen, mimicking pneumothorax, treated successfully with surgical intervention.

## Background

Diaphragmatic hernia is a rare life-threatening condition affecting 3–5 % of all the trauma patients admitted to the hospital [1, 2]. The male to female ratio is about 4:1, mostly presenting in the third decade of life. It results from herniation of visceral contents into the thoracic cavity through a defect in the diaphragm which is either congenital or caused by rupture following trauma. Although acute dyspnea with marked respiratory distress is the most common symptom observed in patients with diaphragmatic hernia, the spectrum of clinical presentation is extremely varied, ranging from no symptom to cardiopulmonary failure and death; therefore, prompt and correct diagnosis with early surgical intervention to definitely treat and prevent recurrence must be undertaken either by abdominal approach (in the acute phase) or transthoracic approach (in the latent phase). Since this condition can mimic pneumothorax both on clinical and radiographic examination, it becomes essential to make the correct diagnosis because approach to treatment of these two conditions differs, and it can be catastrophic if a wrong diagnosis is made and an intercostal drainage tube is inserted. The aim of this report is to highlight that diaphragmatic hernia is an important cause of acute severe breathlessness with respiratory distress.

## Case presentation

**Fig. 1:**
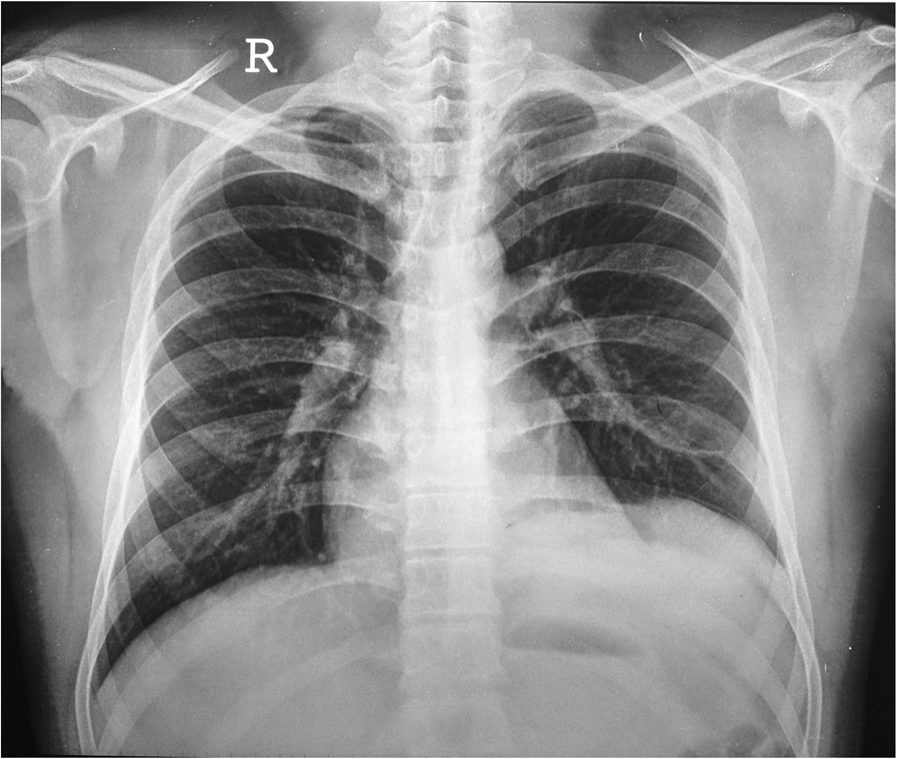
X-ray chest showing raised left dome of the diaphragm

**Fig. 2:**
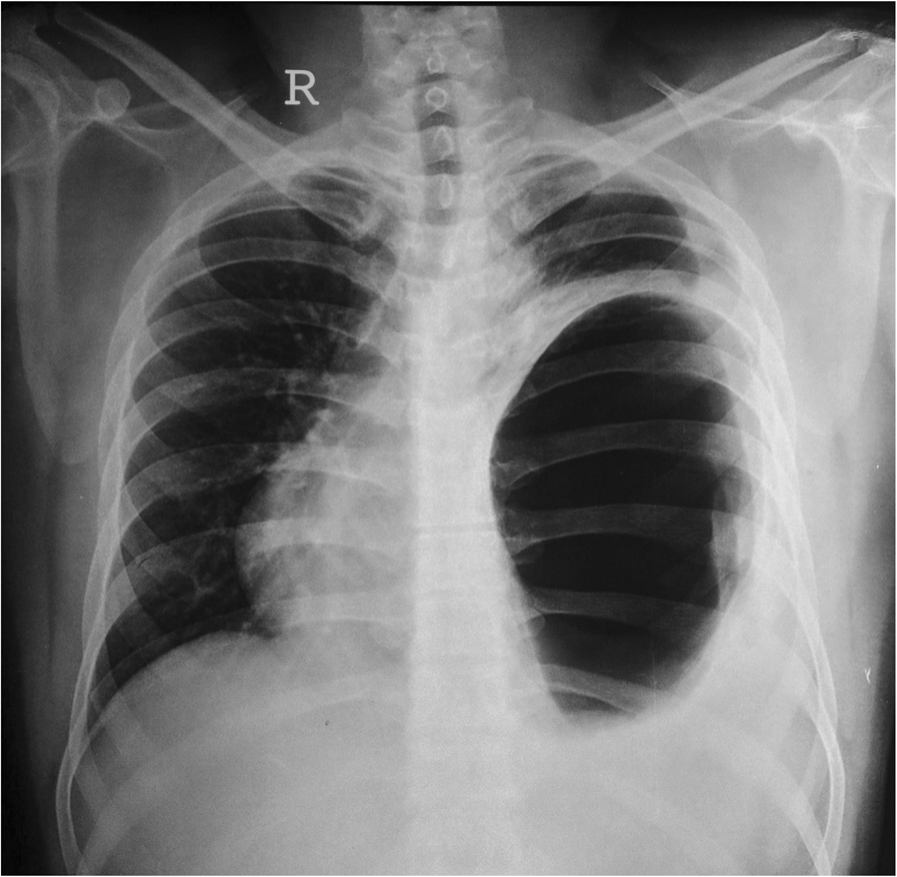
X-ray chest showing encysted hyper-translucence shadow covering the whole left hemi-thorax, with mediastinal shift to the right

**Fig. 3:**
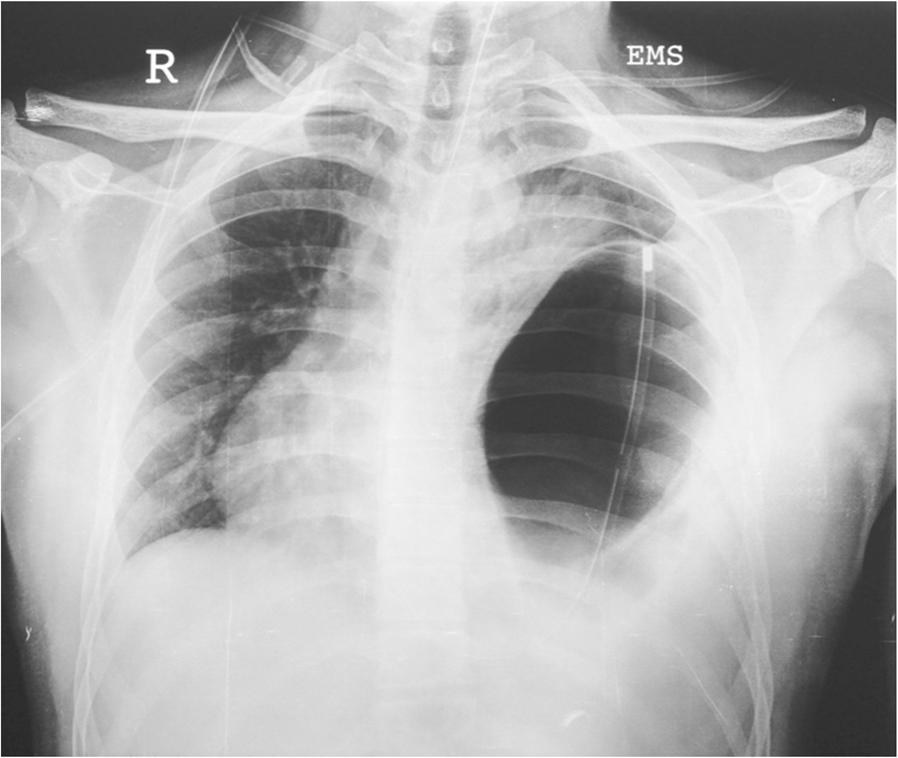
X-ray chest showing encysted hyper-translucence shadow covering the whole left hemi-thorax with nasogastric tube in situ and mediastinal shift to the right

**Fig. 4:**
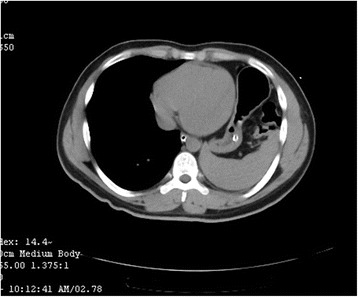
CT scan chest which revealed defect in the left diaphragm posteriorly, consistent with the diagnosis of diaphragmatic rupture with resultant herniation of the stomach, splenic flexure of the colon, spleen, omental fat, descending colon, left-sided transverse colon, bowel, mesentery and in the left hemi-thorax

**Fig. 5:**
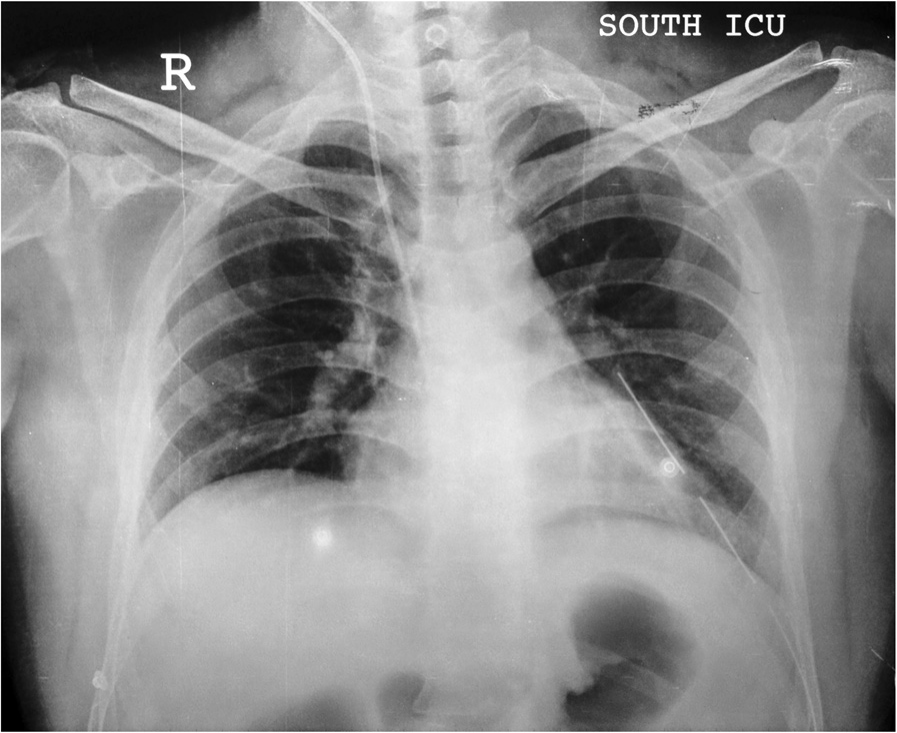
X-ray chest showing full expansion of the left lung with IJV catheter and ICD tube in situ

**Fig. 6:**
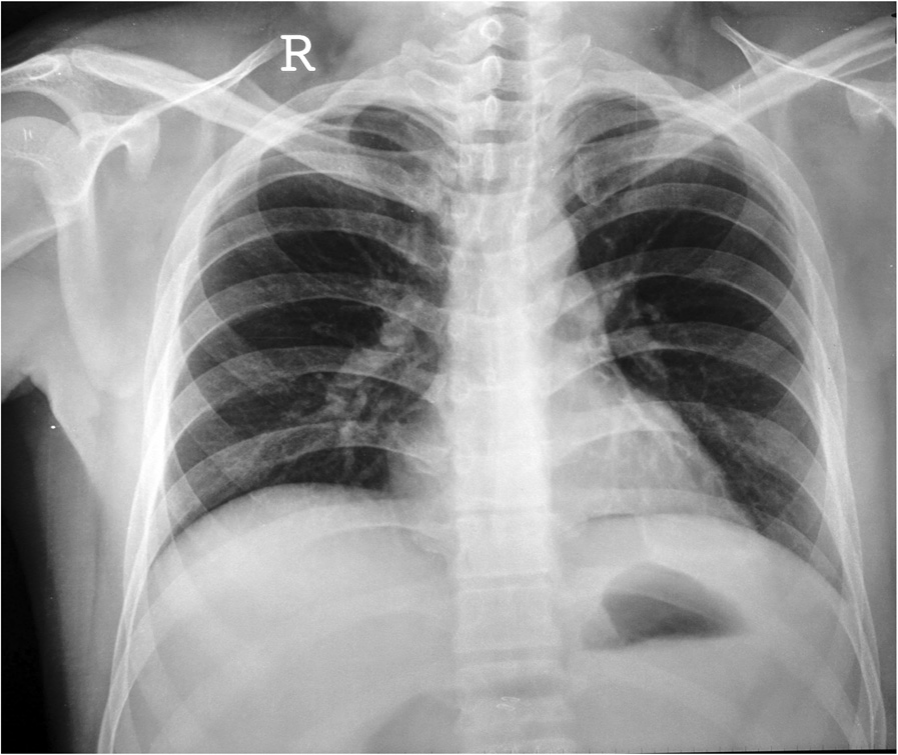
X-ray chest showing full expansion of the left lung on discharge

A 21-year-old male student presented with history of sudden onset of breathlessness, left-sided chest pain radiating to the upper abdomen with nausea, vomiting and hiccup of 4-h duration. There was no history of cough, fever and hemoptysis or other constitutional symptoms. He had history of blunt compressive trauma to the chest 15 days back. Initial chest X-ray (Fig. 1) showed raised left hemi-diaphragm, so he was treated conservatively. He had no history of smoking, alcohol intake or any major illness in the past. On examination, he was fully conscious, oriented in time, place and person and of average build, moderately nourished with height of 165 cm and weight of 55 kg. He was afebrile, and his general physical examination was unremarkable with pulse rate of 112/min, blood pressure 112/68 mmHg, respiratory rate 22/min and saturation was 85 % on room air. Pallor, icterus, cyanosis, clubbing and pedal oedema were not present; J.V.O. not appreciated. Chest examination revealed fullness of intercostal spaces, diminished movements with a resonant note, absent breath sounds and vocal resonance on the left side. Arterial blood gas analysis on 6 l oxygen showed respiratory alkalosis with mild hypoxemia. A clinical diagnosis of left-sided pneumothorax was made. X-ray chest (Fig. 2) revealed a well-defined encysted hyper-translucent opacity covering the whole left hemi-thorax with shift of mediastinum and heart to the right side suggestive of encysted pneumothorax. In view of persisting vomiting and hiccup, a nasogastric tube was inserted resulting in a large gush of air. Subsequent X-ray chest (Fig. 3) showed a nasogastric tube in the left hemi-thorax; possibility of diaphragmatic hernia was considered confirmed by CT scan of the chest (Fig. 4) which revealed a posterior left diaphragmatic defect with resultant herniation of the stomach, splenic flexure of the colon, spleen, omental fat, descending colon, left-sided transverse colon, bowel and mesentery into the left hemi-thorax. He underwent laparotomy which confirmed a large diaphragmatic hernia in the left side postero-lateral region with herniation of the spleen, splenic flexure of the colon and proximal stomach into the thoracic cavity. Reduction of the contents was easily possible, and closure of the defect was done. Further reinforcement was done using a polypropylene mesh. Post-operative course of the patient was uneventful. Follow up X-ray chest (Figs. 5 and 6) showed full expansion of the left lung.

### Discussion

The diaphragm is the major muscle of respiration. Diaphragmatic excursion and chest wall expansion increases the negative intra-thoracic pressure required for inhalation. The sequelae from diaphragmatic rupture and subsequent herniation of intra-abdominal contents are associated with significant morbidity and mortality. Diaphragmatic hernia can be divided in two categories: (1) Congenital hernias: occur because of embryological defects in the diaphragm, which the patient presented early in life; however, a subset of adults may present with congenital diaphragmatic hernia undetected during childhood. (2) Acquired hernias: Stem from all types of trauma with blunt forces seen in the majority of cases. Diaphragmatic hernias require a high level of suspicion as patients can be asymptomatic in up to 53 % from blunt trauma and 44 % from penetrating trauma. Chest X-ray detects only 33 % of hernias when interpreted by a trauma team leader at an initial evaluation. Of all the patients admitted for trauma, 3–5 % have diaphragmatic hernia [1, 3], with male to female ratio of about 4:1, mostly presenting in the third decade of life. Approximately 0.8 to 1.6 % patients with blunt trauma sustain a rupture of the diaphragm accounting for 75 % diaphragmatic hernias, out of which 69 % are left sided, 24 % are right sided and 15 % are bilateral. The left rupture is more common than the right (68.5 % vs 24.2 %) due to hepatic protection and increased strength of the right hemi-diaphragm. Children have equal rates of rupture on both sides due to laxity of liver attachments [4]. The most common cause of blunt trauma is motor vehicle accident, whereas penetrating injuries result from gunshot or stab wounds. Other causes include labour in women with prior diaphragmatic hernia repair [5, 6] and barotrauma during underwater dive in patients with history of Nissen fundoplications [7]. The probable mechanisms for rupture from blunt injuries are (1) shearing of stretched membrane, (2) avulsion of the diaphragm from the points of attachments and (3) sudden increase in the trans diaphragmatic pleuro-peritoneal gradient; the resting pressure difference between pleural (−5 to −10 cm H2O) and peritoneal (+1 to +10 cm H2O) cavities rises to 100 cm H2O with large cough and does not injure the diaphragm. Forces transmitted to the abdomen from blunt trauma can raise the pressure gradient to 1000 cm H2O. The pathophysiology of diaphragmatic hernia includes circulatory and respiratory depression secondary to decreased function of the diaphragm. Herniation of intra-abdominal contents into the thorax leads to pulmonary compression, shifting of mediastinum and cardiac compromise [5]. Clinical presentation includes marked respiratory distress, decreased breath sounds on the affected side, auscultation of bowel sounds in the chest, palpable abdominal contents upon insertion of a chest tube, paradoxical movements of the abdomen with breathing and abdomen being less full on palpation. Initial chest radiography detects 73 % of traumatic diaphragmatic hernias with an additional 25 % found on subsequent film [8]. Chest radiographic findings include [8] (1) abdominal contents in the thorax with or without signs of focal constriction (collar sign), (2) nasogastric tube seen in the thorax, (3) elevated hemi-diaphragm (>4 cm higher on left) and (4) distortion of the diaphragmatic margin. CT chest findings include [8, 9] (1) direct visualization of injuries, (2) segmental diaphragm non-visualization, (3) diffuse thoracic herniation of the viscera, (4) peri-diaphragmatic active contrast extravasation and (5) collar sign. CT scan of the chest has 14–82 % sensitivity with 87 % specificity while sensitivity increased to 71–100 % with helical CT. The left side has higher sensitivity than the right. Ultrasonography scan [10] (focussed assessment with sonography for trauma-FAST) reported decreased movements of the diaphragm, especially in patients who are on ventilator support. Diagnostic laparoscopy and/or video-assisted thoracoscopic surgery (VATS) is indicated in stable patients if isolated diaphragmatic tear is suspected. For traumatic rupture, initial resuscitation is done according to ATLS protocol with airway control being the most important treatment followed by surgical intervention (once the patient’s condition stabilizes) which depends on the timing of diagnosis. In the acute phase of trauma, abdominal approach is preferred while transthoracic approach is preferred in latent phase because the patient often has adhesion to intra-thoracic organs. However, high incidence of concomitant injuries requires emergency exploration in most cases. Early exploration should be done to ensure viability of the herniated contents; repair of the defect should be done preferably reinforced using a polypropylene mesh. Laparoscopic surgery [11, 12] is performed to assess diaphragmatic integrity; it is a minimally invasive procedure by which the diaphragm can be directly visualized to know whether an injury had occurred or not. The diaphragm can be repaired easily by laparoscopic technique [11, 12] in the absence of intra-abdominal injuries. The laparoscopy is helpful in penetrating thoracic and flank injuries with intra-peritoneal penetration. Follow-up and surveillance is important for recurrence which is reported to be very low though small defect have been reported at the repairing site. Outcome and prognosis is good in isolated diaphragmatic injuries without long-term disability; overall mortality has been reported to be in the range of 5.5–51 % with associated complicated intra-abdominal injuries. This case had a history of compressive blunt trauma to the chest, chest pain, and breathlessness and was initially diagnosed as pneumothorax; however, it was confirmed on nasogastric tube insertion and follow-up chest X-ray and CT scan as diaphragmatic hernia improved after laparotomy, with full expansion of the lung. The message here is that the possibility of diaphragmatic hernia should be kept in mind in patients coming to the emergency department with history of breathlessness under respiratory distress.

## Conclusions

Diaphragmatic hernia is one of the causes for emergency department visit of the patient with breathlessness under respiratory distress. The probable causes of diaphragmatic rupture are either congenital or trauma (blunt or penetrating). Chest radiography is the standard diagnostic tool in such cases. Laparotomy is the standard surgical approach for acute phase of trauma. The generally accepted protocol in acute setting is that a diaphragmatic rupture is approached using a celiotomy in view of the presence of intra-abdominal injuries rather than thoracic injuries (84 % vs. 53 %) [13]. For a long standing diaphragmatic injury and subsequent diaphragmatic hernia, a transthoracic or thoracoabdominal approach is preferred because trans-abdominal route is difficult to approach. The case under discussion presents in acute phase so it was treated with laparotomy and reduction of the abdominal contents followed by repair of the diaphragmatic injuries.

## Consent

Patient’s consent was taken.

## References

[1] Turhan K, MacKay O, Caken A (2008). Traumatic diaphragmatic rupture. Eur J Cardiothoracic Surgery.

[2] Cameron JL (2008). Diaphragmatic injury In: Current Surgical Therapy.

[3] Naunheim KS (1998). Adult presentation of unusual diaphragmatic hernias. Chest Surgery Clini NAM.

[4] Mansour KA (1997). Trauma to the diaphragm. Chest Surg Clin N Am.

[5] Jacobs R, Honore PM, Hosseinpour N (2012). Sudden cardiac arrest during pregnancy a rare complication of acquired maternal diaphragmatic hernia. Acta Clin Belg.

[6] Hamoudi D, Bouderka MA, Benissa N, Harti A (2004). Diaphragmatic rupture during labour. Int J Obstet Anesth.

[7] Hayden JD, Davies JB, Martin IG (1988). Diaphragmatic rupture resulting from gastrointestinal barotrauma in a scuba diver. Br J Sports Med.

[8] Sliker CW (2006). Imaging of diaphragm injuries. Radiol Clin North Am.

[9] Desir A, Ghage B (2012). CT of blunt diaphragmatic rupture. Radiographic.

[10] Blaivas M, Brannam L, Hawkins M, Lyon M, Sriram K (2004). Bedside emergency ultrasonographic diagnosis of diaphragmatic rupture in blunt abdominal trauma. Am J Emerg Med.

[11] Xenakis S, Lasthiotkis K, Andreou A, Chrysos E, Chalkiadakis G (2014). Laparoscopic repair of post traumatic diaphragmatic rupture. Report of three cases. Int J Surg case report.

[12] Ahemad N, Whelan J, Chari V, Churg R (2005). The contribution of laparoscopy in evaluation of penetrating abdominal wounds. J Am Coll Surg.

[13] Hanna WC, Ferri LE, Fata P, Razek T, Mulder DS (2008). The current status of traumatic diaphragmatic injury: lessons learned from 105 patients over 13 years. Ann Thoracic Surg.

